# Geochemistry data of trachyandesite of Ulindakonda vent agglomerate of Gadwal greenstone belt of eastern Dharwar Craton, India

**DOI:** 10.1016/j.dib.2023.109275

**Published:** 2023-05-27

**Authors:** N.L.R. Rajeswari Salumuri, N. Dhanamjaya Rao Edupuganti, Sandeep Kumar Kudipudi, Leela Praveen Kandikuppa, Pavan Kumar Medisetty, Abraham Jaydeep Veeravalli

**Affiliations:** Department of Geology, Andhra University, Visakhapatnam 530003, Andhra Pradesh, India

**Keywords:** Trachyandesite, Vent agglomerate, Geochemistry, Gadwal greenstone belt, Tectonic setting

## Abstract

Ulindakonda vent agglomerate of Neo-archean Gadwal Greenstone Belt in the Eastern Dharwar Craton (EDC), is located partly in the Kurnool district of Andhra Pradesh and partly in Jogulamba Gadwal district of Telangana state, India. Trachyandesite occurs as matrix of the agglomerate and at some places it exhibits massive and interbedded nature with granodiorite that mainly occurs as sub-rounded clasts in the agglomerate reflecting magma mixing and mingling. The rock is dotted with small dark specks of a ferromagnesian mineral which often shows a well-developed cleavage surface. The size of grains ranges from medium to fine-grained. Petrographically it is dominated by feldspars and mafic minerals like hornblende and biotite as accessory minerals and quartz in minor amounts. Further, titanite, allanite, carbonate and epidote occur as phenocrysts. Consertal texture is seen between amphibole and quartz, sieve texture also observed in plagioclase feldspar. The percentage of SiO_2_ ranges from 49.84 to 62.92%, TiO_2_ grades from 0.51 to 2.46%, Al_2_O_3_ varies from 11.43 to 15.99%, FeO^T^ ranges from 5.88 to 18.28%, MnO grades from 0.07 to 0.14%, MgO varies from 1.27 to 4.95%, CaO shows variation from 2.58 to 7.62%, Na_2_O grades from 2.56 to 4.84%, K_2_O shows variation from 1.66 to 4.87%, P_2_O_5_ varies from 0.30 to 0.80% and LOI grades from 0.67 to 1.93%. In the primitive mantle-normalized spidergrams, all the samples of trachyandesitic matrix are depleted in high field strength elements (HFSE; Nb, Ti, Zr, Hf and Ta) but are enriched in large ion lithophile elements (LILE; Cs, Rb, Ba, Sr, U, K and Pb). The chondrite normalized REE pattern of trachyandesitic matrix show moderately fractionated LREE (La/Sm_N_= 2.44-4.45, La/Yb_N_= 5.85-23.29) with negligible negative Eu anomaly (0.71-0.91) and flat HREE (Gd/Yb_N_=1.99-3.30) showing the normalized values are >10. The Ulindakonda trachyandesitic samples are plotted in the field of calc-alkaline basalts (CAB) and in the island/ volcanic arc in the tectonic discrimination diagram.

**Specifications Table**

**Table:** 

Subject	Earth Science (Geology)
Specific subject area	Petrography and Geochemistry
Type of data	Table Image Figure
How the data were acquired	Analysis of samples by using XRF and HRICP-MS
Data format	Raw Analyzed
Description of data collection	Field evidences, petrography, geochemical data and tectonics were determined
Data source location	Vent agglomerate of Ulindakonda area, Gadwal Greenstone Belt, Eastern Dharwar Craton, India
Data accessibility	Repository name: [Mendeley Data] Geochemistry of trachyandesite of Ulindakonda vent agglomerate of Gadwal Greenstone Belt of Eastern Dharwar Craton, India Data identification number: 10.17632/56pk6cttym.2 Direct URL to data: https://data.mendeley.com/datasets/56pk6cttym/2 Cite this data set: Salumuri, Rajeswari (2023), “Geochemistry of trachyandesite of Ulindakonda vent agglomerate of Gadwal Greenstone Belt of Eastern Dharwar Craton, India”, Mendeley Data, V2, doi:10.17632/56pk6cttym.2

## Value of the Data


 

• This data consists of the tectonic context, petrography, geochemistry of the trachyandesite of Ulindakonda vent agglomerate, and the geological map of the Gadwal Greenstone Belt.
• This is significant because the Ulindakonda vent agglomerate reflects all the features of vent agglomerate of Archean age. Further, it also exhibits the geochemical characters of an intermediate volcanics (trachyandesite).
• This data may be utilized to gain a better understanding of the geochemical characteristics of the trachyandesite of the Ulindakonda vent agglomerate of the Gadwal Greenstone Belt of the Eastern Dharwar Craton.
• The information is beneficial for advancing research and correlating with the generation of andesitic magmas during subduction.

## 1 Objective

The prime objective of this work is to bring out geochemistry of the trachyandesite that forms the matrix and constitutes a major lithological unit of Ulindakonda vent agglomerate. Further, it is intended to pinpoint the conditions in which volcanic and plutonic rocks are interbedded and forms a lithological unit of agglomerate. The said details will help in the identification of the tectono-environment of the formation of the unit that will be useful in the correlation of the Eastern Greenstone Belts.

## 2 Data Description

**Fig. 1:**
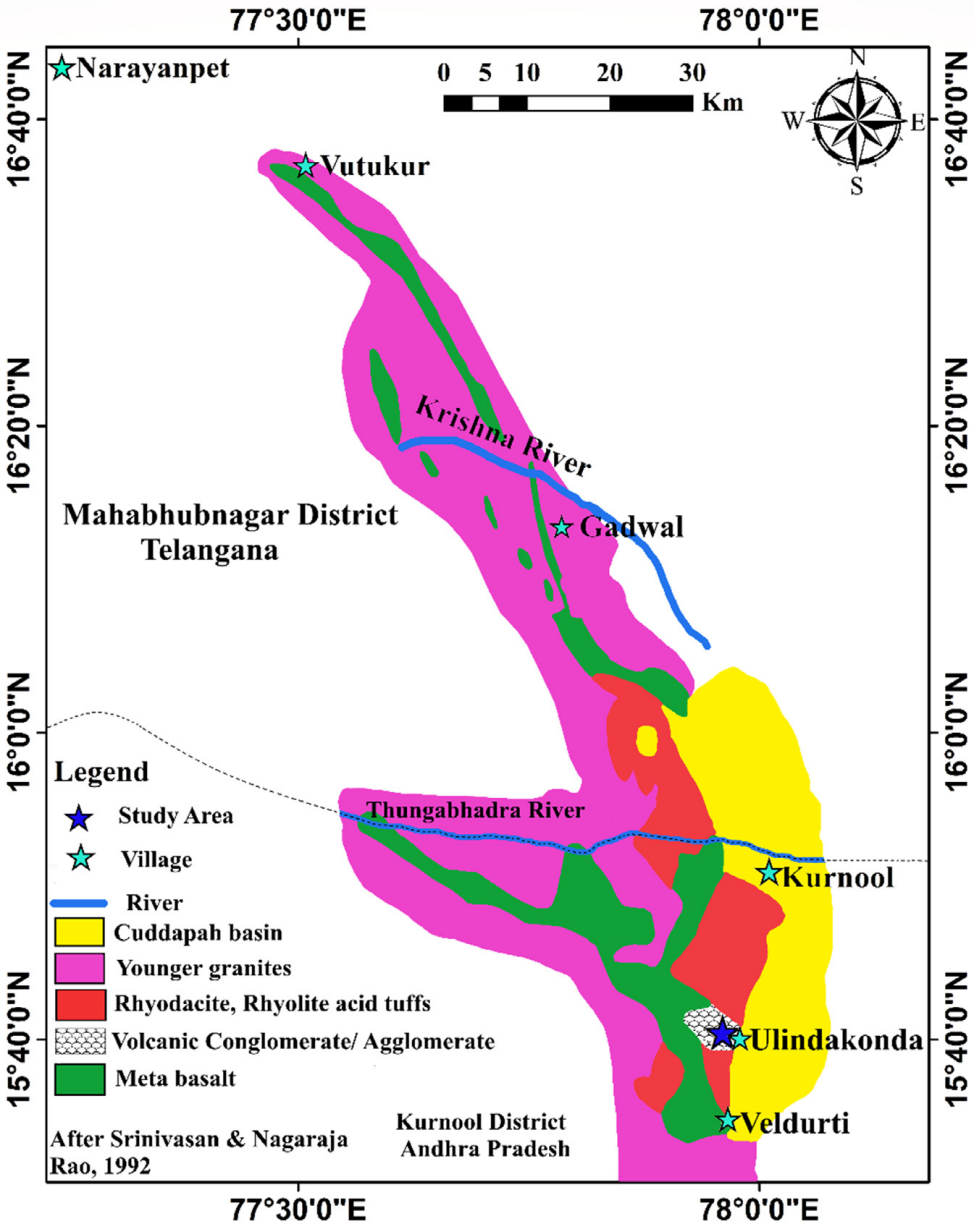
Generalized Geological map of Gadwal Greenstone Belt showing the location of vent agglomerate in Ulindakonda [1].

**Fig. 2:**
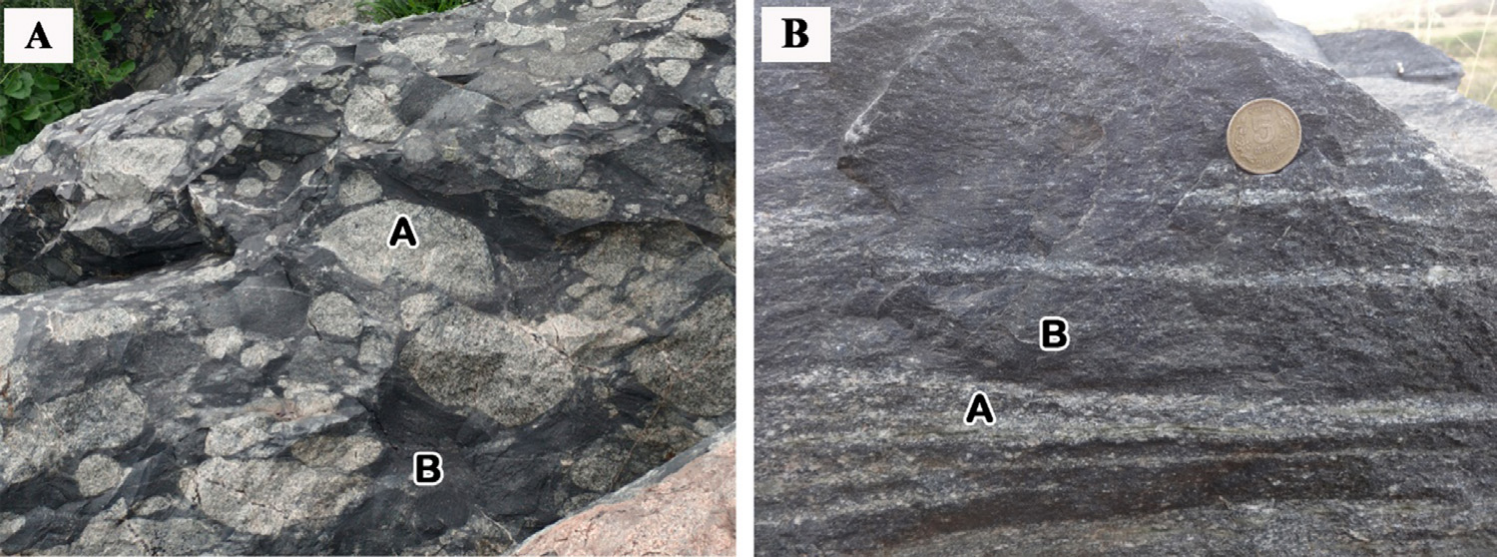
Field photographs showing a) well-rounded granodiorite clasts set in a matrix of trachyandesite, b) banding of granodiorite and trachyandesite; A- Granodiorite, B- Trachyandesite.

**Fig. 3:**
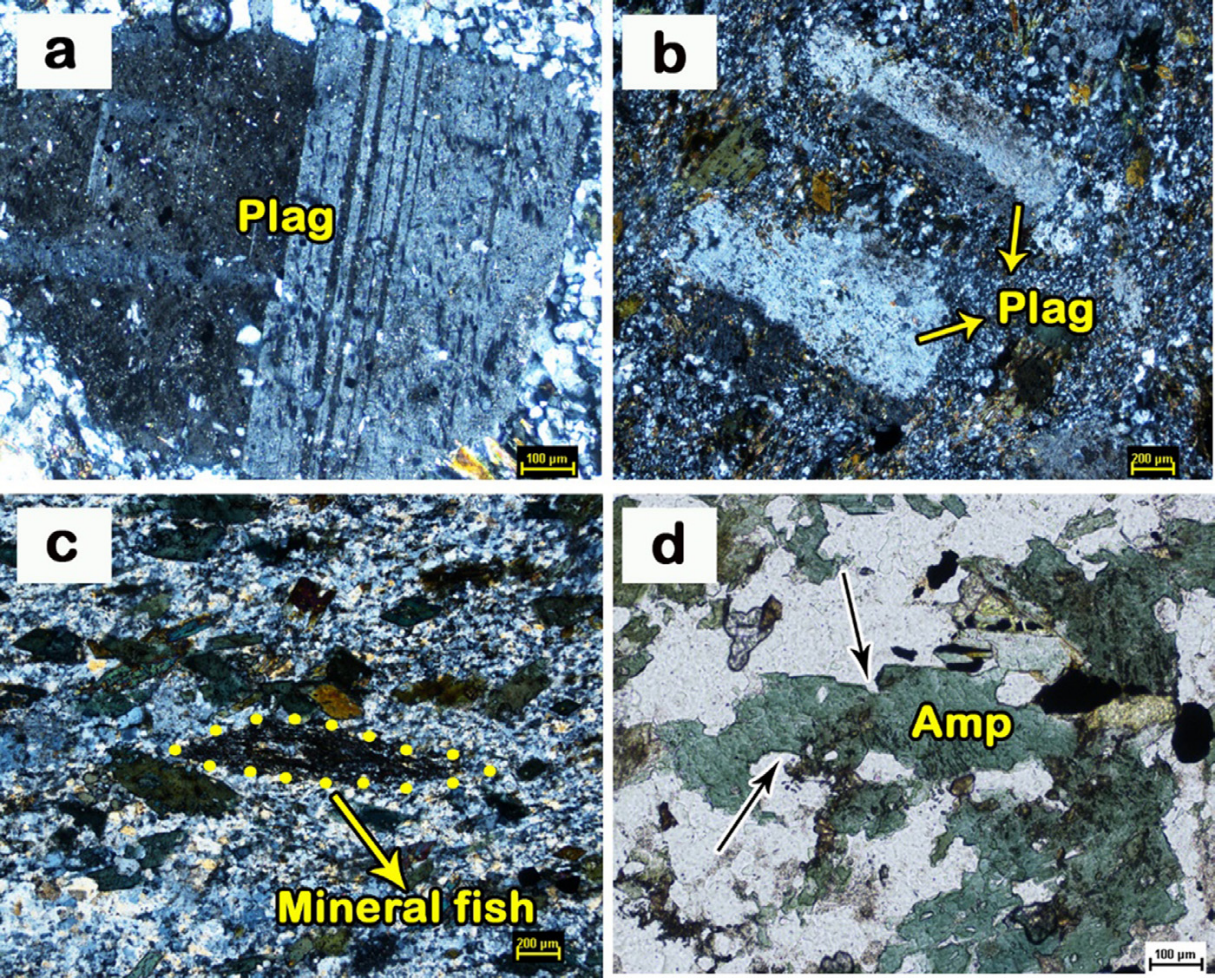
Microphotographs of trachyandesite showing a) sieve texture reflected by plagioclase, b) porphyroclastic texture exhibited by plagioclase, c) mineral fish and d) consertal texture between quartz and amphibole; Plag- plagioclase, Amp- amphibole.

**Fig. 4:**
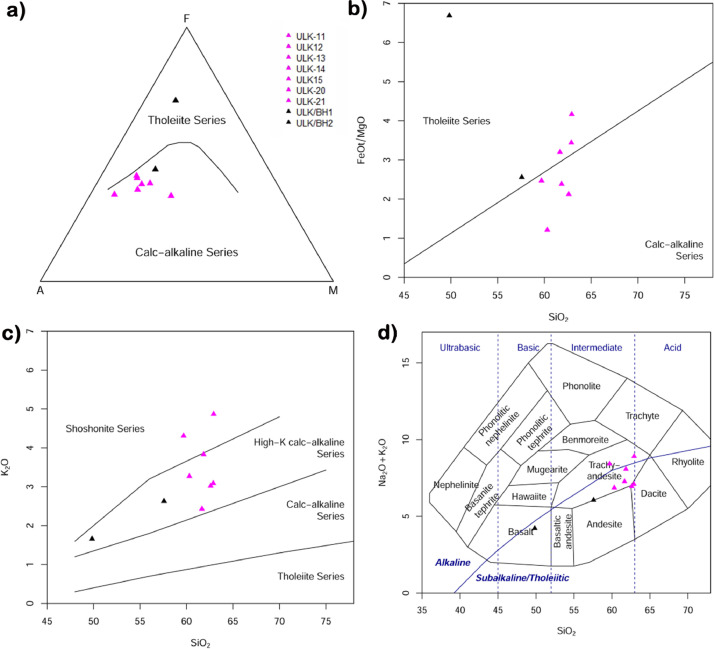
a) AFM diagram showing calc-alkaline nature [2], b) SiO_2_ versus FeO*/MgO reflecting calc-alkaline and tholeiite series [3], c) SiO_2_ versus K_2_O showing high-K calc-alkaline and shoshonite series [4], d) TAS-Total alkalies (Na_2_O+K_2_O) versus SiO_2_ diagram reflecting trachyandesite and basalt [5] (ULK-Ulindakonda; BH-Borehole).

**Fig. 5:**
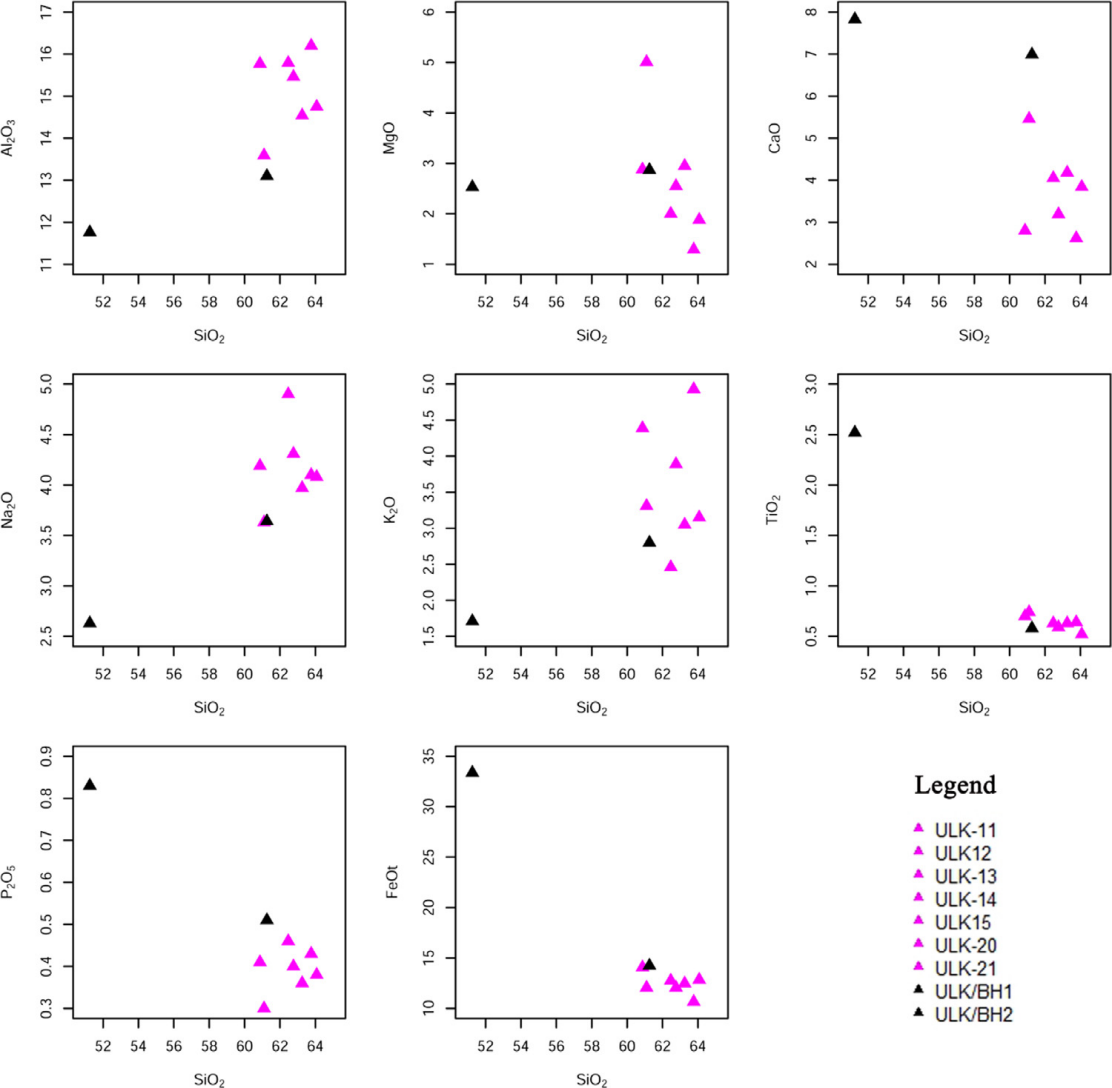
SiO_2_ versus major oxides for Harker variation diagrams of trachyandesite [8].

**Fig. 6:**
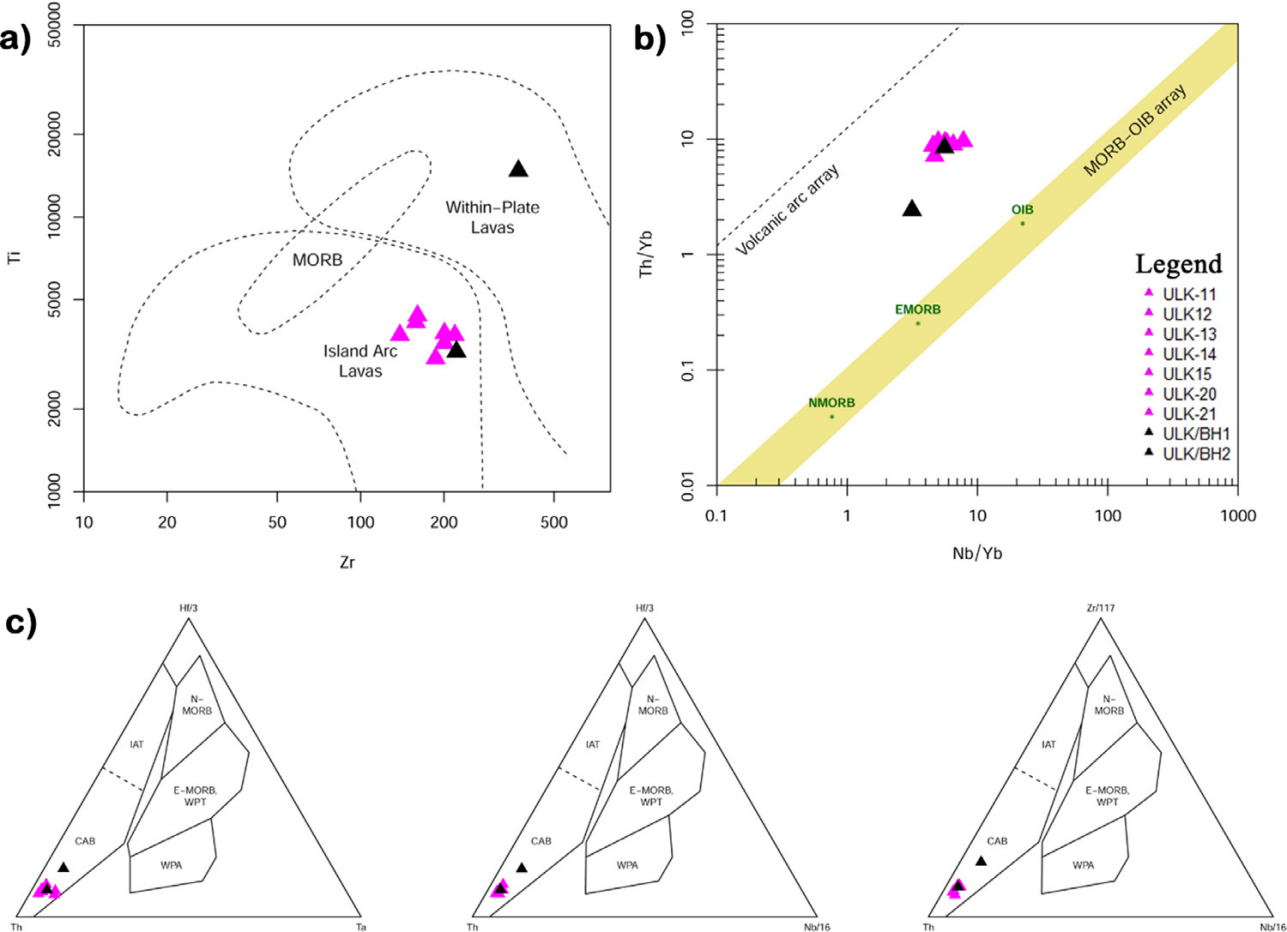
Tectonic discrimination diagrams of trachyandesite a) Zr vs Ti reflecting island arc lavas, b) Nb/Yb vs Th/Yb diagram showing volcanic arc environment, c) Hf/3-Th/Ta, Hf/3-Th-Nb/16 and Zr/117-Th-Nb/16 ternary diagrams showing calc-alkaline basalts (CAB).

**Fig. 7:**
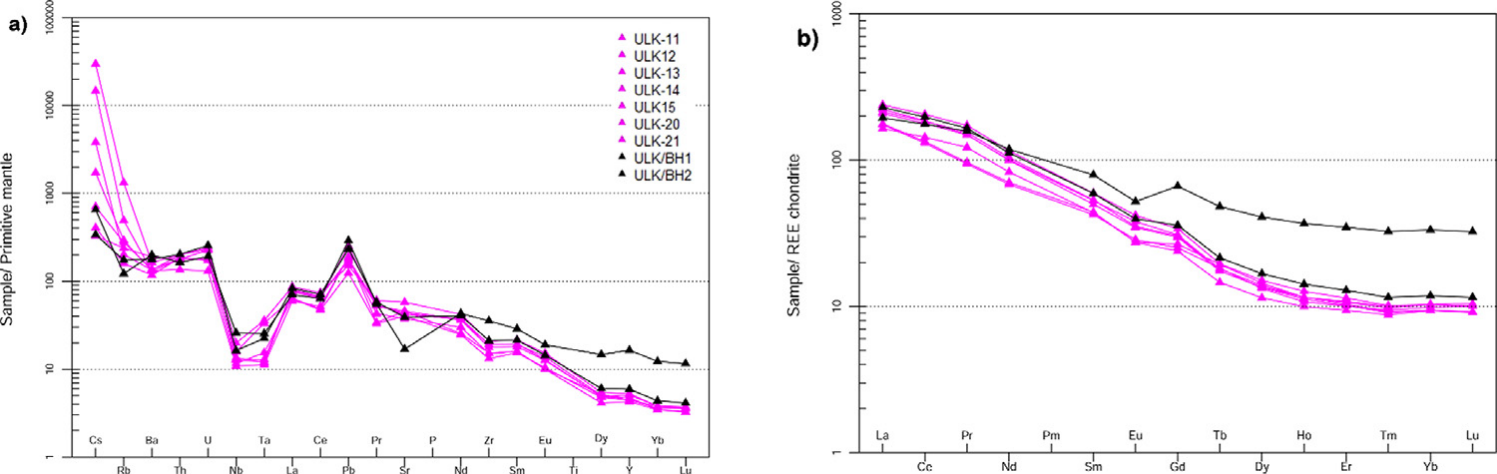
a) Primitive mantle normalized multi-element diagram [11], b) Chondrite normalized REE diagram [12].

The data set of this article provides detailed geological map of Gadwal Greenstone Belt reflecting the study area (Fig. 1), field photographs (Fig. 2), microphotographs (Fig. 3), major oxide and trace elements data table (Table 1) and various geochemical plots (Figs. 4-7).

**Table 1:** Major oxides, trace and REE elements of trachyandesitic matrix and borehole samples of Ulindakonda Formation. The chips from the drilled borehole, last two samples (ULK/BH1 and ULK/BH2) have been collected to study the rock type in the central part of the vent.

SI No.	1	2	3	4	5	6	7	8	9
Sample	ULK11	ULK12	ULK13	ULK14	ULK15	ULK20	ULK21	ULK/BH1	ULK/BH2

Major oxides (Wt.%)									
SiO_2_	62.92	60.31	62.88	61.66	62.60	61.84	59.69	57.58	49.84
Al_2_O_3_	15.99	13.41	14.47	15.59	14.39	15.23	15.46	12.31	11.43
Fe_2_O_3_	5.88	6.66	7.07	7.04	6.89	6.65	7.74	7.67	18.28
MnO	0.07	0.10	0.11	0.10	0.11	0.09	0.11	0.09	0.14
MgO	1.27	4.95	1.85	1.98	2.92	2.51	2.82	2.70	2.46
CaO	2.58	5.39	3.77	4.00	4.14	3.15	2.75	6.57	7.62
Na_2_O	4.04	3.59	4.01	4.84	3.93	4.25	4.11	3.43	2.56
K_2_O	4.87	3.27	3.09	2.43	3.02	3.83	4.31	2.63	1.66
TiO2	0.63	0.73	0.51	0.62	0.62	0.58	0.69	0.54	2.46
P2O5	0.42	0.30	0.38	0.46	0.35	0.40	0.40	0.48	0.80
LOI	0.67	1.10	0.86	0.86	0.92	1.08	1.10	4.52	1.93
Sum	99.34	99.81	99.00	99.57	99.89	99.61	99.16	98.51	99.17
Trace and rare earth elements (ppm)									
Sc	20.67	15.39	14.23	16.55	11.12	18.20	20.00	18.11	35.38
V	173.64	140.13	129.70	149.58	101.61	142.63	121.29	157.07	410.44
Cr	259.76	41.67	220.01	199.64	37.10	216.00	255.84	141.65	128.44
Co	19.94	23.42	24.95	20.06	18.94	21.24	21.95	24.19	65.01
Ni	72.20	15.01	59.21	51.00	56.19	58.09	53.65	59.54	93.27
Cu	46.64	59.55	46.04	30.64	72.15	28.22	28.90	53.23	133.44
Zn	60.37	93.76	89.27	55.60	92.67	81.20	61.12	33.44	83.78
Ga	24.63	19.29	22.57	29.14	20.84	23.43	18.40	21.58	25.26
Rb	143.32	96.00	121.41	796.80	295.76	166.07	175.27	104.88	73.29
Sr	911.71	906.07	870.33	1149.85	798.56	831.62	745.06	776.97	336.67
Y	20.26	18.94	21.76	22.64	19.14	20.38	18.38	25.44	70.86
Zr	200.81	160.55	186.59	218.99	138.68	200.58	158.49	221.96	371.73
Nb	8.91	7.65	8.38	13.03	10.41	8.60	7.14	10.73	17.02
Cs	6.89	8.52	80.14	626.09	308.77	14.80	36.18	7.10	13.71
Ba	1250.30	780.22	888.01	1082.89	858.10	913.26	872.09	1174.53	1319.10
Hf	4.52	4.22	4.14	4.96	3.73	4.44	4.10	5.10	8.13
Ta	0.43	0.57	0.44	1.32	1.23	0.47	0.41	0.83	0.93
Pb	38.02	28.72	25.87	22.61	18.63	27.14	28.09	34.90	43.56
Th	14.16	14.73	15.99	16.03	14.35	14.36	10.84	16.28	13.10
U	4.60	3.47	4.84	4.67	3.62	4.52	2.62	5.10	3.84
La	48.99	41.59	52.3	56.10	41.15	50.40	38.66	53.92	45.66
Ce	107.73	80.97	112.3	124.15	78.88	111.40	86.51	119.07	106.30
Pr	13.24	8.60	13.33	15.40	8.39	13.79	10.89	14.72	14.08
Nd	45.61	31.83	45.16	52.56	30.90	47.12	37.56	50.79	53.72
Sm	7.7	6.49	7.35	8.75	6.23	7.86	6.43	8.71	11.67
Eu	2.12	1.53	1.93	2.34	1.60	1.97	1.53	2.21	2.91
Gd	6.06	5.24	5.84	6.67	4.95	5.97	4.70	7.04	13.07
Tb	0.65	0.70	0.63	0.71	0.66	0.63	0.53	0.78	1.74
Dy	3.38	3.50	3.29	3.65	3.21	3.27	2.77	4.06	9.90
Ho	0.64	0.64	0.64	0.70	0.59	0.62	0.55	0.79	2.04
Er	1.64	1.71	1.69	1.81	1.59	1.61	1.49	2.04	5.51
Tm	0.22	0.24	0.23	0.24	0.22	0.21	0.21	0.27	0.78
Yb	1.53	1.68	1.67	1.67	1.59	1.53	1.52	1.92	5.41
Lu	0.22	0.25	0.24	0.24	0.24	0.22	0.22	0.28	0.78

### 2.1 Field Observations and Petrography

Gadwal Greenstone Belt (GGB) is one of the Precambrian supracrustal belts, collectively known as Dharwar Greenstone Belts located in the Eastern Dharwar Craton (EDC) of South India. This belt extends from Veldurti in Kurnool district of Andhra Pradesh in the South to Narayanpet in Mahbubnagar district of the Telangana State in the North covering an area of 85 km along the strike and has ∼ 1-15 km width. Ulindakonda vent agglomerate is considered as a unit of the Gadwal Greenstone Belt. In the South, near Ulindakonda, vent agglomerate consisting of bombs/ blocks (> 64 mm) of granodiorite, basalt and andesite are set in a matrix of trachyandesite. Stratigraphically Gadwal Greenstone Belt is divided into a) lower Sangala Formation, comprising metabasalts and amphibolites b) upper Ulindakonda Formation, consisting of vent agglomerate [1]. Along with the vent agglomerate, boninites have also been reported [6]. Felsic volcanic flows, including dacites, rhyodacites, rhyolite and adakites and calcsilicate unis, are observed in the South [7]. Srinivasan and Nagaraja Rao (1992) [1] carried out extensive work on geological mapping, geochemistry and proposed lithostratigraphy of the belt that has been modified in this paper.

The vast area covered by Ulindakonda vent agglomerate reflects a flat or gently undulating topography. There are nearly 8 outcrops in and around the Ulindakonda village that show positive relief and a few smaller outcrops are located near Chetlamallapuram village (towards north) without any positive relief. The field setup reflects a spherical shape with a crater-like depression with extensive volcanic material points to a vent. The approximate limit of extension of the Ulindakonda vent agglomerate is confined by basalt towards the west. The outcrops are divided into, i) outcrop with sub-rounded to rounded granodiorite clasts set in a trachyandesitic matrix (Fig. 2a) and ii) outcrops with stratification of granodiorite and trachyandesite (Fig. 2b).

The study of petrography was carried out in the Petrological laboratories of Geological Survey of India, Hyderabad. Trachyandesite consists of plagioclase and orthoclase as essential minerals whereas quartz, magnetite, titanite, allanite, hornblende, biotite, carbonate and epidote as accessory minerals. Sieve texture is observed in plagioclase feldspar of trachyandesitic matrix (Fig. 3a). The presence of large mineral grains set in a matrix of smaller crushed grains indicates porphyroclastic texture (Fig. 3b). Further, the porphyries have an elongate shape with monoclinic symmetry resembling mineral fish (Fig. 3c). Intergranular consertal texture between amphibole and quartz is also noticed (Fig. 3d).

### 2.2 Geochemistry

Major and minor oxides were determined by using XRF, nearly 34 trace elements including REE were analysed using HRICP-MS. The XRF data (Table- 1) shows that SiO_2_ ranges from 49.84 to 62.92%, TiO_2_ grades from 0.51 to 2.46%, Al_2_O_3_ varies from 11.43 to 15.99%, FeO^T^ ranges from 5.88 to 18.28%, MnO grades from 0.07 to 0.14%, MgO varies from 1.27 to 4.95%, CaO shows variation from 2.58 to 7.62%, Na_2_O grades from 2.56 to 4.84%, K_2_O shows variation from 1.66 to 4.87%, P_2_O_5_ varies from 0.30 to 0.80% and LOI grades from 0.67 to 1.93%. In the AFM ternary diagram (Fig. 4a) [2], all the samples of trachyandesitic matrix occupy the calc-alkaline field except for one sample i.e., borehole (BH2) sample occupy tholeiitic series as it is mainly basaltic with a slight increase in iron content. In the FeO*/MgO versus SiO_2_ (Fig. 4b) [3], the samples ULK-11, ULK-13, ULK-14, ULK-BH1 and ULK-BH2 occupy the tholeiitic field due to the increase in FeO*/MgO content whereas, the samples ULK-20 & ULK-21 occupy the calc-alkaline field with low iron content. In the binary plot of SiO_2_ versus K_2_O (Fig. 4c) [4] most of the samples are plotted in the high-K-calc-alkaline series except two samples (ULK-11 and ULK-21) plotted in shoshonite series due to high K_2_O. The TAS-Total alkalies (Na_2_O+K_2_O) versus SiO_2_ (Fig. 4d) [5], plot in the field of trachyandesite and basalt.

The Harker diagram of trachyandesitic matrix (Fig. 5), indicate a cluster, but a broadly negative trend in the case of Fe_2_O_3_, P_2_O_5_, and TiO_2_. The Na_2_O gives a cluster nature and K_2_O value is much more i.e., 4.87 wt% as compared with the standards of K_2_O value i.e., 1.8 wt%, and exhibits a positive trend. The MgO also reflects broadly negative character and MnO points to the linear character. The FeO/MgO versus SiO_2_ reflects a positive trend.

In the primitive mantle-normalized spidergrams (Fig. 7a), all the trachyandesite samples are depleted in high field strength elements (HFSE; Nb, Ti, Zr, Hf and Ta) but are enriched in large ion lithophile elements (LILE; Cs, Rb, Ba, Sr, U, K, and Pb) are characterized by subduction related magmas. They show moderately fractionated LREE with negligible negative Eu anomaly and flat HREE (Fig. 7b). Most of the subducted island arc magmas are calc-alkaline in nature viz., basaltic andesites and andesites. The basaltic andesites is generally higher in Al2O3 and enriched in Ba, Sr, REE and LILE elements and are also low in Nb and Ta. Zr versus Ti binary plot (Fig. 6a) [9] suggested that the trachyandesites erupted in a volcanic arc environment. In Hf/3-Th-Ta, Hf/3-TH-Nb/16 and Zr/117-Th-Nb/16 ternary diagrams (Fig. 6c) [10] of trachyandesitic samples are plotted in the field of calc-alkaline basalts (CAB).

## 3 Experimental Design, Materials and Methods

The rock samples of trachyandesite were collected from the fresh outcrops and un-weathered specimens were selected for thin section-preparation and representative samples for whole rock geochemical analysis. The methods of investigations involve laboratory studies pertaining to petrography of varied rock types. Detailed petrographic studies of all the rock groups were studied by using Petrological microscope. The representative samples were powdered to ∼ 250 mesh by using agate mortar and subjected to major and trace element analysis including rare earth elements determination using XRF and HRICP-MS techniques. Major elements (SiO_2_, TiO_2_, Al_2_O_3_, CaO, Fe_2_O_3_, MgO, MnO, Na_2_O, K_2_O and P_2_O_5_) have been analysed using the pressed pellets on X-Ray Florescence Spectrometer (XRF; Phillips MAGIX PRO Model 2440). Trace (Sc, V, Cr, Co, Ni, Cu, Zn, Ga, Rb, Sr, Y, Zr, Nb, Ba, Cs, Hf, Ta, Pb, U, Th) and rare earth (La, Ce, Pr, Nd, Sm, Eu, Gd, Tb, Dy, Ho, Er, Tm, Yb, Lu) elements concentrations were determined by using High-Resolution Inductively Coupled Mass Spectrometry (HRICP-MS; Model Attom, Nu Instruments, UK) at CSIR-National Geophysical Research Institute (NGRI), India. The REE and trace elements data were normalized using Chondrite [12] and Primitive mantle [11]. Regardless of the initial state of the diluted sample, the temperature was fixed at 5°C to ensure homogeneity in sample viscosity and solution density, ensuring approximately consistent spinning of the solution inside the spray chamber. The sample dissolutions were carried out in Savillex pressure decomposition containers (60 mL; Savillex Corporation, Minnetonka, MN, USA). For sample preparation, electronic grade HF, distilled HNO_3_ and HCl, and analytical reagent (AR) grade HClO_4_ were utilized. The wet chemical approach was used to dissolve a few selected rock samples. In Savillex screw top containers, a test piece (0.05 g) of each sample was collected, and 10 mL of an acid combination (7:3 HF-HNO_3_) was added. Then, as an internal reference, 5 mL of a 1 ng/mL 103Rh solution were added to each vessel. They were well stirred, then shut firmly and heated to around 140°C for 48 hours. In order to assure thorough elimination of HF from the mixture, the jars were then opened, and the contents were evaporated at 200°C to almost dryness with a few drops of HClO_4_. It was further dissolved by adding 10 mL of 1:1 HNO_3_, and 250 mL of Milli-Q de-ionized water was added to the mixture, which was then kept in HDPE bottles. To eliminate mistakes caused by reagent and handling, a few procedural blanks were also made using the sample batch using the same methodology as above. The scanning of specific ions was carried out in jump-wiggle mode (similar to peak hopping), allowing precise measurement of the analytes of interest. A typical Meinhard nebulizer with a cyclonic spray chamber integrated in a Peltier cooling system served as the sample introduction.

## Ethics Statement

This article does not contain any studies with human or animal subjects.

## CRediT authorship contribution statement

**N.L.R. Rajeswari Salumuri:** Conceptualization, Methodology, Software, Validation, Formal analysis, Investigation, Resources, Data curation, Writing – original draft, Writing – review & editing, Visualization, Supervision, Project administration, Funding acquisition. **N. Dhanamjaya Rao Edupuganti:** Supervision. **Sandeep Kumar Kudipudi:** Software, Investigation, Resources. **Leela Praveen Kandikuppa:** Resources. **Pavan Kumar Medisetty:** Investigation, Resources. **Abraham Jaydeep Veeravalli:** Resources, Visualization.

## Declaration of Interests

The authors declare that they have no known competing financial interests or personal relationships that could have appeared to influence the work reported in this paper.

## Data Availability

Geochemistry data of trachyandesite of Ulindakonda vent agglomerate of Gadwal Greenstone Belt of Eastern Dharwar Craton, India (Original data) (Mendeley Data).
